# Exploring oncogenes for renal clear cell carcinoma based on G protein-coupled receptor-associated genes

**DOI:** 10.1007/s12672-023-00795-z

**Published:** 2023-10-10

**Authors:** Chengcun Zhu, Zhou Sun, Jie Wang, Xiangdi Meng, Zhaosen Ma, Rui Guo, Jiqiang Niu, Lisa Jia Tran, Jing Zhang, Tianxiao Jiang, Yunfei Liu, Fangdie Ye, Baoluo Ma

**Affiliations:** 1 Department of Urology, The First People’s Hospital of Jiangxia District, Wuhan, 430200 Hubei China; 2 Department of Urology, Xiangyang Central Hospital, Affiliated Hospital of Hubei University of Arts and Science, Xiangyang, Hubei China; 3 Department of Urology, China-Japan Union Hospital of Jilin University, Changchun, 130000 Jilin China; 4 Department of Urology, The Second People’s Hospital of Meishan City, Meishan, Sichuan China; 5 Department of General, Visceral, and Transplant Surgery, Ludwig-Maximilians-University Munich, 81377 Munich, Germany; 6 Division of Basic Biomedical Sciences, The University of South Dakota Sanford School of Medicine, Vermillion, SD 57069 USA; 7 Department of Urology, Huashan Hospital, Fudan University, Shanghai, China

**Keywords:** Clear cell renal cell carcinoma, G protein-coupled receptors, TCGA, Transcriptome profiling, Single cell RNA sequencing, Survival analysis, Tumor microenvironment

## Abstract

G protein-coupled receptors (GPCRs) are a class of receptors on cell membranes that regulate various biological processes in cells, such as cell proliferation, differentiation, migration, apoptosis, and metabolism, by interacting with G proteins. However, the role of G protein-coupled receptors in predicting the prognosis of renal clear cell carcinoma is still unknown. The transcriptome data and clinical profiles of renal clear cell carcinoma patients, were downloaded from TCGA databases, and the validation group data were downloaded from number GSE167573, including 63 tumor samples and 14 normal samples. Single-cell RNA sequencing data were downloaded from the GEO database, No. GSE152938 and selected samples were used for GSEA enrichment analysis, WGCNA subgroup analysis, single-cell data analysis, and mutation analysis to explore the role of G protein-coupled receptor-related genes in the diagnosis and prognosis of renal clear cell carcinoma and to verify their reliability with cellular experiments. Finally, this study establishes a disease model based on G protein-coupled receptor-related genes, which may help to propose targeted therapeutic regimens in different strata of renal cell carcinoma patients.Author names: Please confirm if the author names are presented accurately and in the correct sequence (given name, middle name/initial, family name). Author: Given name [Lisa Jia] Last name [Tran].It's ok!

## Introduction

Renal cell carcinoma (RCC), also known as renal cancer, represents the most prevalent renal cancerous and the third-most prevalent adult urological tumor. It is caused by cells of the renal tubular epithelial cells in the renal parenchyma [1, 2]. According to a compilation of large-scale healthcare data surveys from 185 countries, 431,288 health care workers were newly diagnosed with kidney tumors in 2020, and 179,368 passed away as a result [3]. The latest survey showed that 76,080 new cases and 13,780 deaths are expected in the United States in 2021 [4]. It accounts for 85% of renal and 2% of all adult malignancies worldwide [5, 6]. Clear cell carcinoma, papillary cell carcinoma, and suspicious cell carcinoma are the three main pathophysiological subtypes of kidney cancer, with clear cell carcinoma becoming the most common [7, 8]. The formation of kidney cancer is related to multiple factors [9]. When a single gene is defective in a cell, it does not kill the cell. However, when two or more genes are defective simultaneously, it can kill the cell. We call this phenomenon a synergistic lethal effect [10]. The early clinical manifestations of kidney cancer are often very insidious and not easily detected.

However, with medical imaging technology’s advancement, the kidney cancer detection rate has increased dramatically. Most of the detected kidney cancers are limited-stage kidney cancers. However, 20–30% of patients still have distant metastases at the time of diagnosis, or metastases occur 1–2 years after surgery, called metastatic renal cell carcinoma (mRCC). mRCC patients have a poor prognosis and related literature. The 5-year survival rate of mRCC patients is less than 10% [11–13]. The main medical treatments for mRCC include cytokine therapy and targeted drug therapy. The essential characteristics of malignant tumors are grouped into 10 categories, and each cancer characteristic corresponds to a specific treatment strategy [9] .

G protein-coupled receptors (GPCRs) are a class of receptor proteins with seven transmembrane helices that play an essential role in biology [14, 15]. G protein-coupled receptor 4 (GPR4) is one of the four receptors of the OGR1 subfamily, which is widely distributed in various human tumor tissue cells and participates in and influences tumorigenesis and development. It was found that malignant tumors can affect the expression of GPR4 and vascular endothelial growth factor (VEGF) and its receptors directly or indirectly due to excessive proliferation, severe local tissue hypoxia, and the formation of an acidic environment [16–18], which affects the microvascular density of tumor tissues and thus the development, infiltration, and metastasis of tumors, and GPR4 plays an essential role in the vascular endothelial cells of angiogenesis. Furthermore, they are involved in related diseases and cancer-related angiogenesis. Related studies have shown that they can influence the tumor microenvironment (TME) and thus the proliferation and migration of cancer cells, contributing to tumor development, invasion, and malignant transformation [19, 20]. Therefore, pathophysiological angiogenesis is a tumor marker associated with poor prognosis in many cancer types [21, 22] .

This study aimed to create an assessment method to evaluate the predictive efficacy of G protein-coupled receptor-related genes on the prognosis of patients with renal clear cell carcinoma. we performed a comprehensive analysis of risk models, including functional enrichment, immune infiltration evaluation, and single-cell sequencing analysis. Our findings reveal a critical role for G protein-coupled receptor-related genes in renal clear cell carcinoma patients. We propose a convenient method to help diagnose and predict survival outcomes in renal clear cell carcinoma patients.

## Materials and methods

### Data resources and data preprocessing

The bulk RNA data were downloaded from XENA-TCGA-KIRC with 607 samples, including 535 patients and 72 control samples. The corresponding clinical data were downloaded from TCGA-GDC, including patients’ gender, age, survival time, survival status, tumor stage grading, etc. The validation group data were downloaded from the GEO database with the number GSE167573, including 63 tumor and 14 normal samples. Single-cell RNA sequencing data were downloaded from the GEO database, number GSE152938, and samples were selected for renal clear cell carcinoma, GSM4630028, and GSM4630029, respectively.

### Construction and optimization of prognostic models

Based on the collected G protein-coupled receptor-related genes (GRPs), multi-factor cox regression analysis and prognostic models were constructed using the survival package and glmnet package, respectively, to select the GRPs associated with the prognosis of renal clear cell carcinoma and the best of them were selected by optimization using the bootstrap method. The samples in the training group were scored based on the GRPs genes associated with the prognosis of renal clear cell carcinoma, and their GRPs correlation scores were calculated, with the median score as the cut-off and those with scores more significant than the median as the high group and those lower than the score as the low group. The immune infiltration in the TCGA samples was calculated using the CIBERSORT method. Those immune cell types (naive B cells, monocytes, activated dendritic cells) that were prognostically relevant were evaluated using the surfeit function and optimized using the bootstrap method to calculate their TME scores, bounded by the median score, with scores more significant than the median being the high subgroups and those with scores lower than the median as low subgroups. The correlation between the high and low subgroups of GRPs and TMEs and the prognosis of renal clear cell carcinoma was assessed separately.

### Pathway enrichment analysis

Differential analysis of GRPs scores and TME scores in high and low groups was performed using the limma package, and enrichment analysis of differential genes was performed based on the KEGG gene set.

### WGCNA subgroup analysis

The hclust function was used to cluster the expression matrix. After removing the discrete subclusters, the pickSoftThreshold function was used to cluster the expression data with a powerVector of 1:30, the blockwiseModules function was used to visualize the clustering results, and the corPvalueStudent function was used to determine the correlation type between the two phenotype scores and the modules. The labeledHeatmap function presents the correlation heat map and selects the modules that correlate with the two phenotype scores.

### Correlation analysis of phenotype scores and prognosis results

Based on the respective prognostic analysis before the two phenotypes, the GRPs high subgroup and TME low subgroup were combined (for the worst prognosis type), the GRPs low subgroup and TME high subgroup were combined (for the best prognosis type), and the intermediate type was defined as mixed. Based on this method to explore the prognosis of patients based on age, gender, and staging classification of the disease, subgroup survival analysis was done and repeated in the validation group Validation.

### Single-cell RNA sequencing data analysis

Based on the seurat package, create seurat objects using the CreateSeuratObject function, select cells expressing between 200 and 4000 genes for subsequent analysis, downscale cluster and visualize the data using the UMAP method, and annotate the cells into 10 different types based on classical cell markers. Expression scoring was performed for each cell type based on GRPs using the AddModuleScore function—cell communication analysis of single-cell data using the cellchat package. Use the online site TIP to calculate immune processes. Use the online tool TIDE to evaluate the effect of immunotherapy on the training set and perform subsequent analysis based on the response to immunotherapy. The genes were grouped according to the immune response gap. Differentially up-and down-regulated genes between the groups were obtained. Shatter plots were drawn using an online tool (https://proteomaps.net/) to observe the functions and pathways of genes associated with immune and prognostic gaps.

### Cell culture

Kidney cancer cell line 786-O, Caki-1-1 cell line was presented by Dr. Chen and supplemented with 10% fetal bovine serum (Invitrogen, USA) at 37 °C in 5% CO_2_.

### Gene interference caused by small interfering RNA

Sangon created the oligonucleotide primers encoding small interfering RNA (siRNA) for genes (Shanghai, China). Kidney cancer cells 786-O and CAKI-1 cells (si-NC group, si-1 group, si-2 group) transfected either with or without specific siRNA against genetic makeup were seeded into microplate at a concentration of 6000 cells per well and incubated for 3 days.

### qRT-PCR detection of RNA expression levels

Total RNA was obtained using a Trizol kit following the manufacturer’s instructions, and RNA purity and concentration were determined using a Nano-300 microspectrophotometer. qRT-PCR was used to detect the expression of target-specific mRNA.

### EDU cell proliferation experiment

The CELL-Light EDU DNA Cell Proliferation Kit (RiboBio, Guangzhou, China, Cat. No. C10310-1) was used for the EDU assay. Kidney cancer cells 786-O, CAKI-1 cells were incubated with 50 µM EDU buffer for 2 h before being fixed in 4% formalin. Then the results were visualized by a fluorescence microscope.

### The transwell assay

Cells have been digested 48 h after transfection, collected in serum-free DMEM medium. The lower chamber was filled with complete medium. The upper chamber was filled with serum-free medium containing cells. After 24 h, the chambers were removed and fixed in paraformaldehyde for 30 min; crystal violet was soiled for 30 min; and the amount of migrated cells was tallied and recorded in 5 fields of each compartment under the microscope.

### Protein expression levels are detected using Western blot

Cells were obtained and lysed on ice with a mixture of RIPA lysate and PMSF, and the total protein of each group of cells was extracted, and the protein content of each group was determined using the BCA kit. sampled proteins were subjected to SDS-PAGE, 250 mA steady flow membrane transfer, and closed with TBST closure solution for 2 h; antibodies against E-cadherin protein, Snail protein, and Vimentin protein were added, overnight at 4 °C, rinsed with TBST the next day, and then HRP-labeled secondary antibody was added.

### Assay for colony formation

The number of colonies formed assessed the ability of clone formation. In 6 cm well plates, 1000 cells were inoculated and incubated at 37 °C. In accordance with the features of each cell line, the medium was changed every 10–15 days. PBS was used to wash the adherent cells twice before they were fixed with 4% paraformaldehyde and soiled with 0.1% crystal violet. Total number of colonies was calculated (50 cells per colony).

### Statistical analysis

R was used for all statistical analyses. Experiments were repeated at least for three times independently. Measured data were represented as the mean ± SD.

## Results

### Construction and optimization of prognostic models

**Fig. 1:**
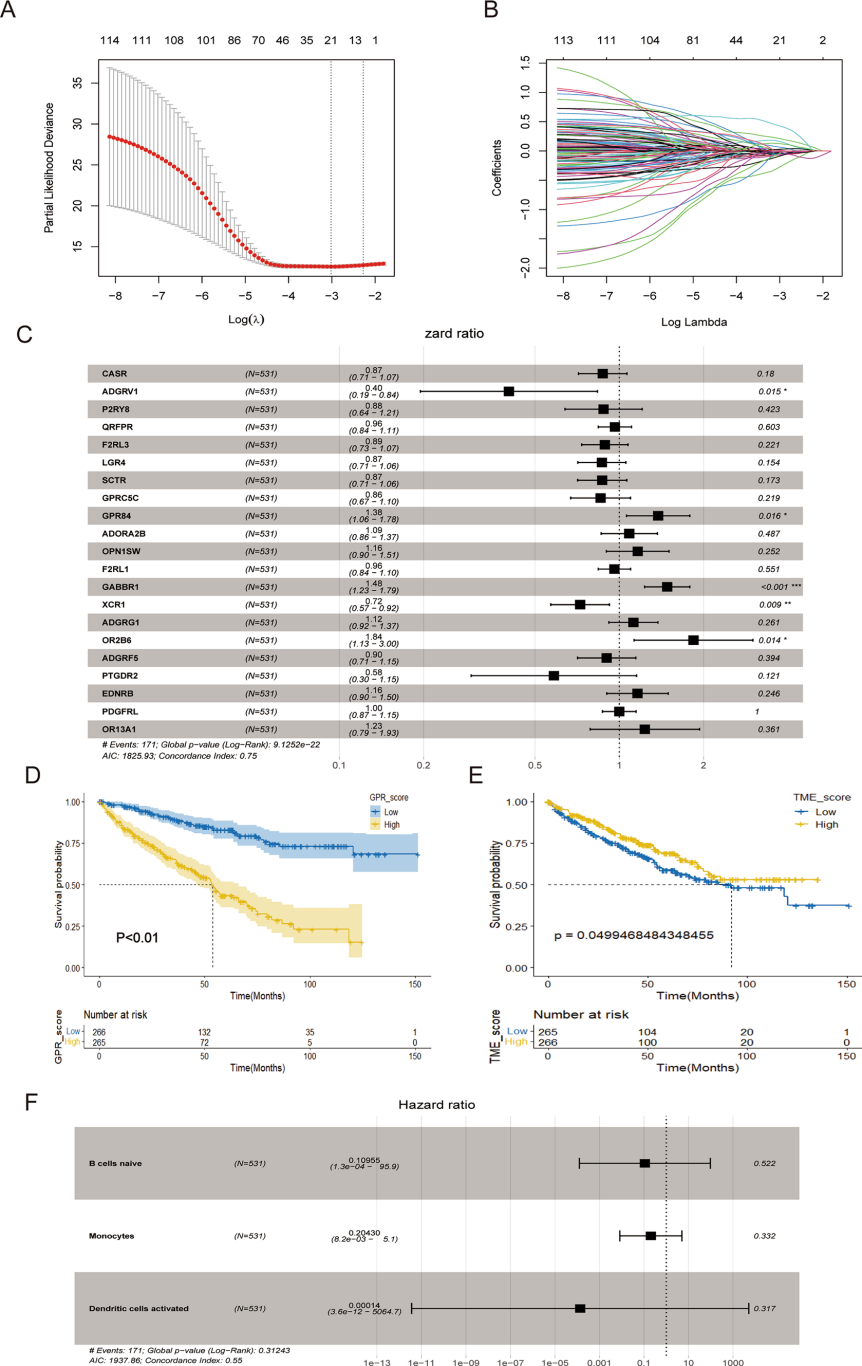
LASSO regression model and results of multi-variate regression analysis. **A** LASSO coefficient distribution of 163 differentially expressed genes associated with prognosis of renal clear cell carcinoma, each curve represents a coefficient, when the tuning parameter changes, non-zero coefficients change with it into the LASSO regression model. **B** cross-validation of selecting the best λ, the red dashed line corresponds to the minimum of the multivariate Cox model when crossed with the best log λ. The two dashed lines indicate the distance minimum one standard deviation. **C** Results of multivariate Cox regression analysis based on the training set. **D** K–M curves based on GPR scores. **E** K–M curves based on TME scores. **F** Forest plots based on immune cells

The univariate Cox regression analysis showed that 163 genes were strongly correlated with the prognosis of patients with renal clear cell carcinoma. In the LASSO analysis, the corresponding coefficients of the identified genes decreased to 0 as logλ changed (Fig. 1A). In the cross-validation, 28 genes reached the minimum value of partial fit deviation, and 28 genes showed some effect with HRs greater than 1, suggesting a positive effect on the development of bladder cancer (Fig. 1B). Multifactorial Cox regression analysis of the training set data yielded 21 genes with GRPs as independent predictors, namely CASR, ADGRV1, P2RY8, QRFPR, F2RL3, LGR4, scTR, GPRC5C, ADORA2B, OPN1sw, F2RL1, GABBR1, XCR1, ADGRG1 OR2B6, ADGRF5, PTGDR2, EDNRB, (Fig. 1C). The training group samples were scored, their GRPs correlation scores were calculated, and the median score was used as the boundary. Those with scores more outstanding than the median were the high GRPs group, and those with scores lower than the median were the low GRPs group, and the predictive analysis showed that the high GRPs group had a worse prognosis than the low GRPs group (Fig. 1D). Using the survfit function to assess those immune cell types associated with prognosis, the results showed that naïve B cells, monocytes, and activated dendritic cells were associated with prognosis. The CIBERSORT method to calculate immune infiltration in TCGA samples was bounded by the median score. Those with scores more remarkable than the medians were the TMEs high subgroup, and those below the score were the TMEs low subgroup, and the prognosis The results of the analysis showed that the TMEs high subgroup had a better prognosis than the TMEs low subgroup (Fig. 1E, F).

### GSEA enrichment analysis

**Fig. 2:**
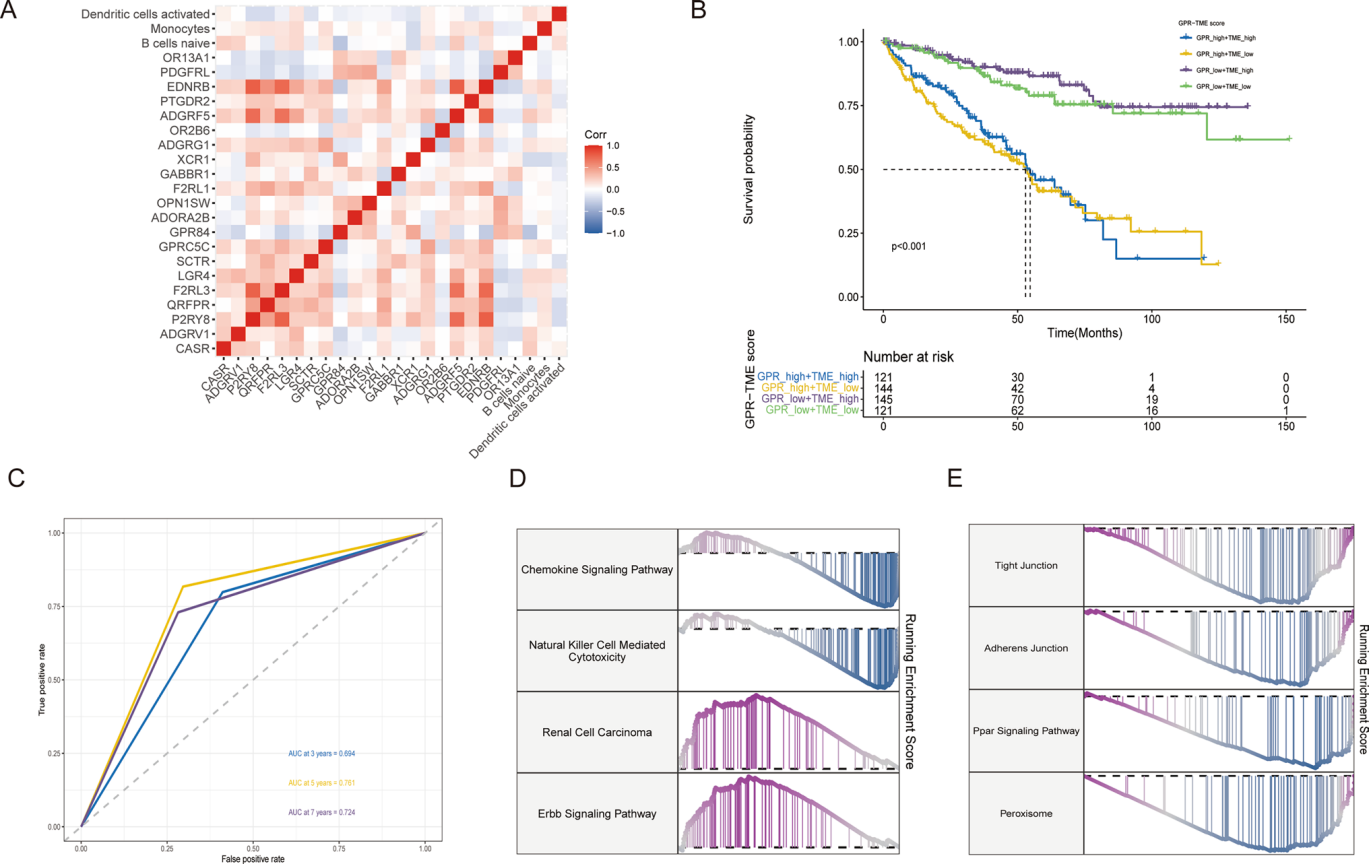
Pathway enrichment analysis. **A** Correlation between 21 GRPs genes. **B** Prognostic analysis of different GRPs and TMEs gene groupings.**C** Prediction model based on GRPs and TMEs gene expression levels (**D**, **E**) regulation pathways in high- and low-risk groups

We further showed the correlation between the 21 GRPs genes by Pearson correlation analysis and plotted the correlations (Fig. 2A). To test the reliability of GRPs and TMEs score in the prognosis of patients with renal clear cell carcinoma, we divided the patients into four gene groups according to the GRPs and TMEs gene expression levels in cancer patients, GRPs high + TMEs high, GRPs high + TMEs low, GRPs low + TMEs high, and GRPs low + TMEs low groups, and the predictive analysis showed that the GRPshigh−TMElow group had the worst prognosis and GRPslow−TMEhigh group had the best prognosis (Fig. 2B), and the AUCs based on the prediction model of GRPs and TMEs gene expression levels at 3, 5 and 7 years were 0.694, 0.761, and 0.724, respectively, all of which were greater than 0.6, indicating an excellent predictive efficacy (Fig. 2C). Differential analysis of GRPs scores and TME scores for high and low groups was performed using the limma package. Enrichment analysis of differential genes was performed. GSEA enrichment analysis showed that the significant differential downregulation pathways for high and low-risk groups were: Chemokine Signaling Pathway, Natural Killer Cell-Mediated Cytotoxicity, Tight Junction, Adherens Junction, Ppar Signaling Pathway, and Peroxisome. The upregulated pathways were the Renal Cell Carcinoma Erbb Signaling Pathway (Fig. 2D, E).

### Differentially expressed genes related to overall survival status of patients with clear cell renal cell carcinoma

**Fig. 3:**
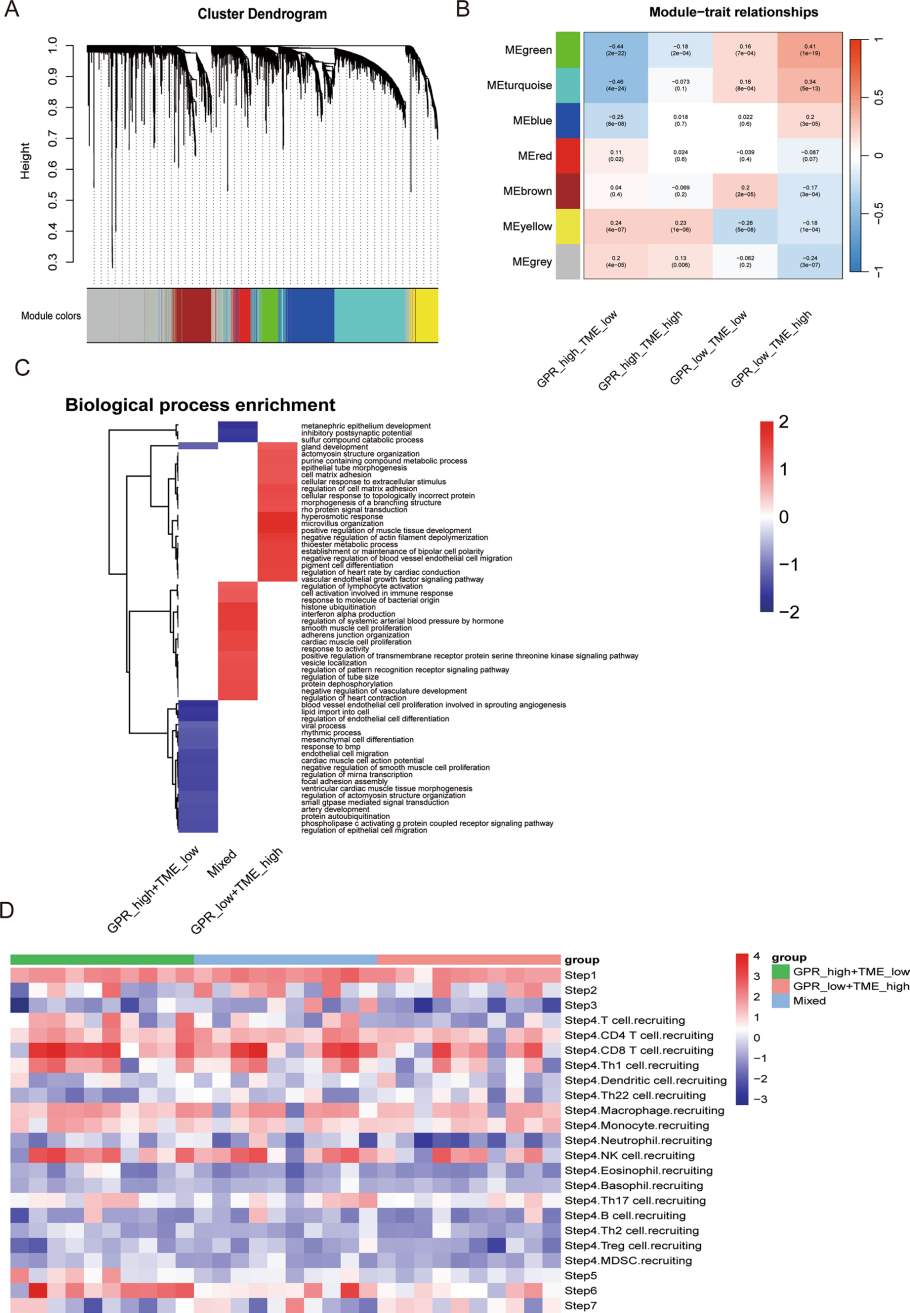
Prognosis-related differentially expressed genes in renal clear cell carcinoma. **A** Gene clustering based on topological overlap matrix, with relatively related genes located on the same or adjacent branches. **B** Correlation of module eigengenes with renal clear cell carcinoma. **C** GO enrichment analysis, **D** different Immune step correlated with subgroups

Gene mapping and clinical characterization data were extracted from 607 samples downloaded from XENA-TCGA-KIRC, including 535 patients and 72 control samples, with a soft threshold of 13. 12 color-coded modules were generated based on topological overlap matrix gene clustering with a dynamic tree cut of module size 30 (Fig. 3A). Overall survival status was correlated with all 7 modules, with turquoise and green modules showing the highest correlation with renal clear cell carcinoma (Fig. 3B). GO enrichment analysis revealed GPR_low + TME_High: enriched in metanephric epithelium development, the inhibitory postsynaptic potential, sulfur compound catabolic process, gland development, and actomyosin structure organization. Mixed: enriched in response to molecule of bacterial origin, histone ubiquitination, interferon alpha production, regulation of sysTMEic arterial blood pressure by hormone. gpr _high + TME_low: enriched in mesenchymal cell differentiation, response to BMP, endothelial cell migration, cardiac muscle cell action potential, and negative regulation of smooth muscle cell proliferation. corPvalueStudent function was used to determine the correlation type between the two phenotype scores and the modules. A correlation heat map was drawn to select the modules correlated with the two phenotype scores (Fig. 3C). The correlation heat map showed that CD8 T, NK, Th1 cell recruiting, and Macrophage recruiting expressions were relatively high in the GPR_high + TME_low group. In the GPR_low + TME_high group, The expression of CD4 T cell recruiting, Th1 cell recruiting, and Macrophage recruiting was relatively high in the Mixed group recruiting expression was relatively high (Fig. 3D).

### Prognosis of renal clear cell carcinoma

**Fig. 4:**
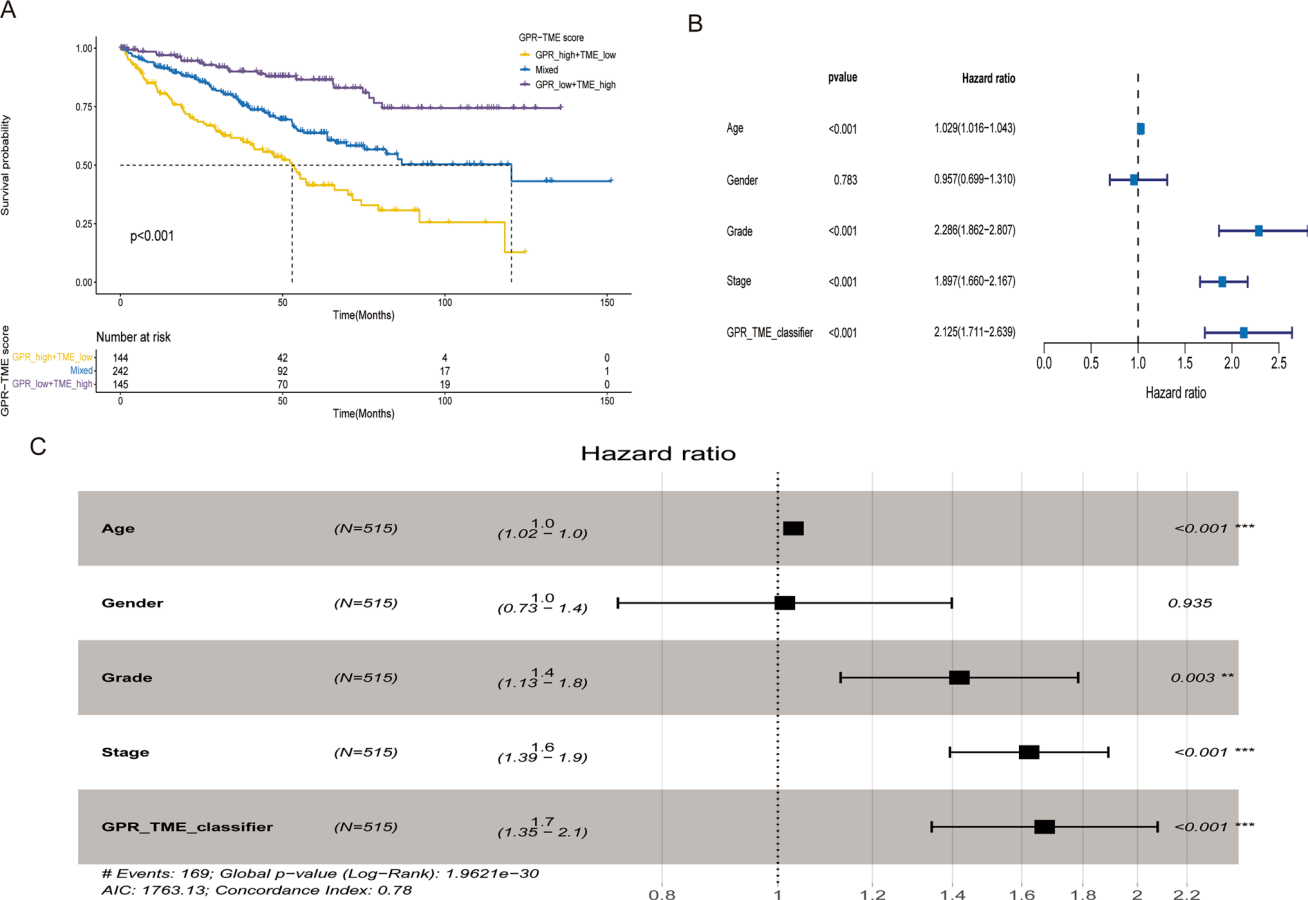
Correlation analysis of subgroup and prognostic outcome. **A** K–M prognostic model for different subgroups of renal clear cell carcinoma. **B**, **C** Regression analysis of age, sex, and disease of renal clear cell carcinoma based on multivariate Cox analysis

**Fig. 5:**
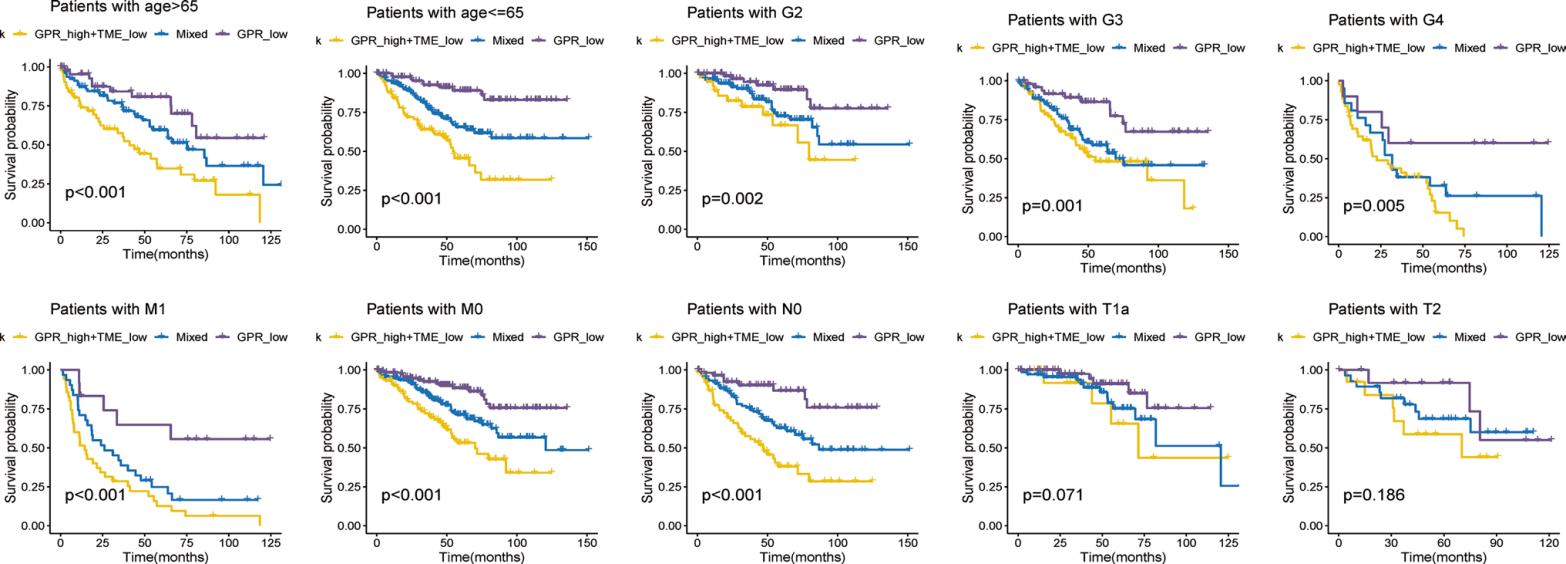
Prognosis of patients with renal clear cell carcinoma based on age, sex, and disease staging classification was explored

The prediction model results showed that the combination of GRPs low grouping and TME high grouping (as the best prognosis type) and the intermediate type defined as mixed. The prognosis of GRPs low grouping + TME high grouping was better, the prognosis of GRPs high grouping + TME low grouping was the worst, and the prognosis of the mixed group was intermediate (Fig. 4A). The results of forest plot analysis showed a correlation between GRPs and TME scores on the prognosis of renal clear cell carcinoma (Fig. 4B). We were repeated in the GSE167573 validation group, and the results were consistent with the verify group (Fig. 4C). Based on different clinical features, the KM curve showed significant difference in prognosis between high-risk group and low-risk group (Fig. 5).

### Single-cell RNA sequencing analysis

**Fig. 6:**
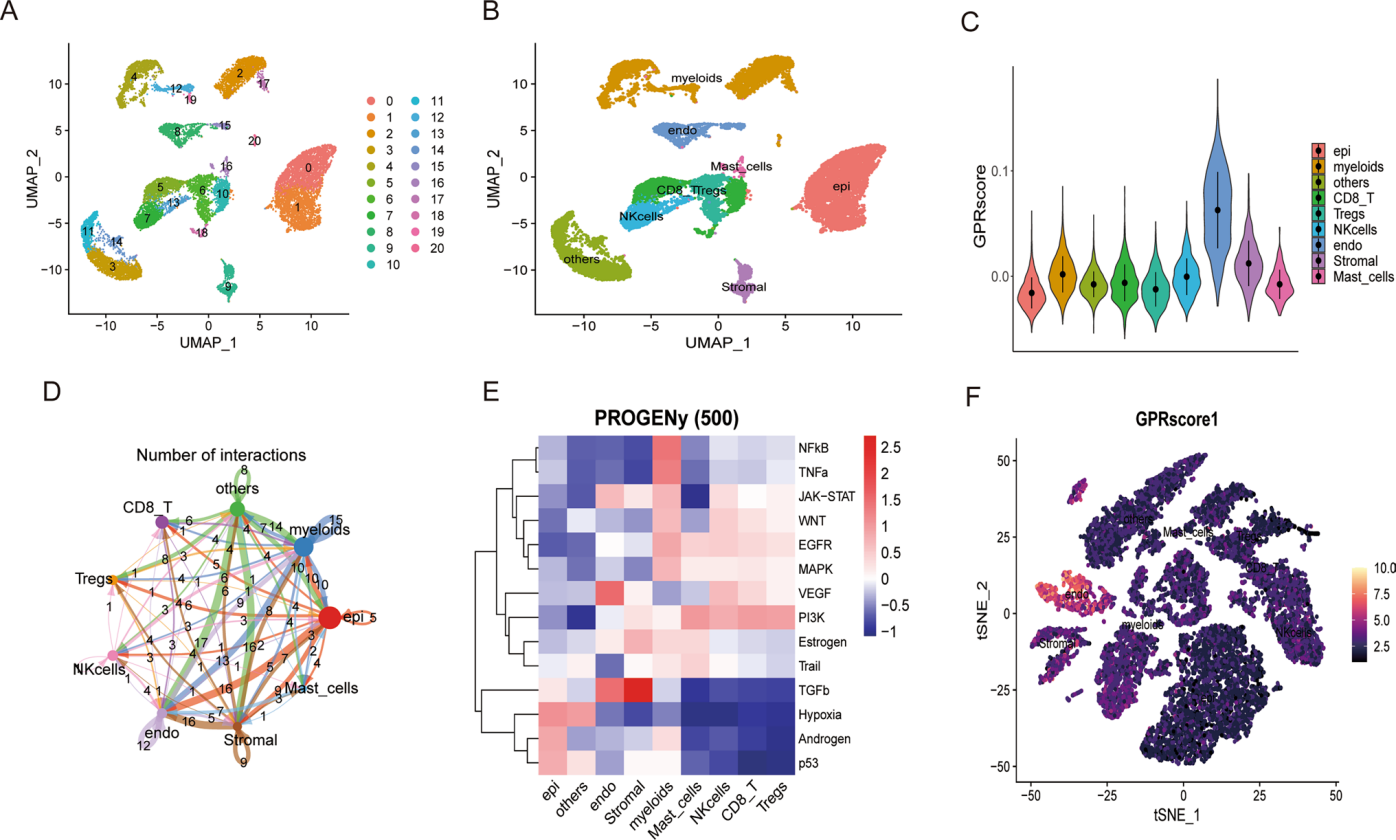
Analysis of single-cell RNA sequencing data for renal clear cell carcinoma. **A**, **B** UMAP was clustered into 21 clusters and divided into 7 cell subgroups. **C** GPR scores of different cell subgroups. **D** Number of intercellular interactions plotted. **E** PROGENy scores. **F** Evaluation of immunotherapy effect

ScRNA.seq was applied to analyze the cellular differences between cancer tissues and normal regional tissues in 2 patients with renal clear cell carcinoma. The single-cell sequencing data were subjected to data normalization, dimensionality reduction analysis, and cluster analysis, and then all cells were divided into 21 cell clusters (Fig. 6A). It included 7 immune cells clusters such as myeloid, endo, Masticells, Nkcells, CD8-T, Tregs, and Stromal (Fig. 6B). The GPRs scores of various immune cell clusters are also shown (Fig. 6C). Cell chat is a database containing receptor-ligand interactions with 2021 validated molecular interactions. cellChat can identify critical features of intercellular communication in a given scRNA-seq dataset and predict potential signaling pathways that are currently studied Differential analysis of intercellular communication indicates that myeloid, endo, Masticells, Nkcells, CD8-T, Tregs, Stromal, and other cell populations interact closely (Fig. 6D). Furthermore, PROGENy scores showed that Masticells, Nkcells, CD8-T, Tregs were positively correlated with activating “NFkB, TNFa, JAK-STAT, WNT, EGFR, MAPK, VEGF, PI3K, Estrogen Trail”. On the contrary, myeloids, endo, and Stromal cells were positively correlated with “TGFb, Hypoxia, Androgen, p53 activation” (Fig. 6E). And to assess the effect of immunotherapy in the training set (Fig. 6F).

### Mutation analysis

**Fig. 7:**
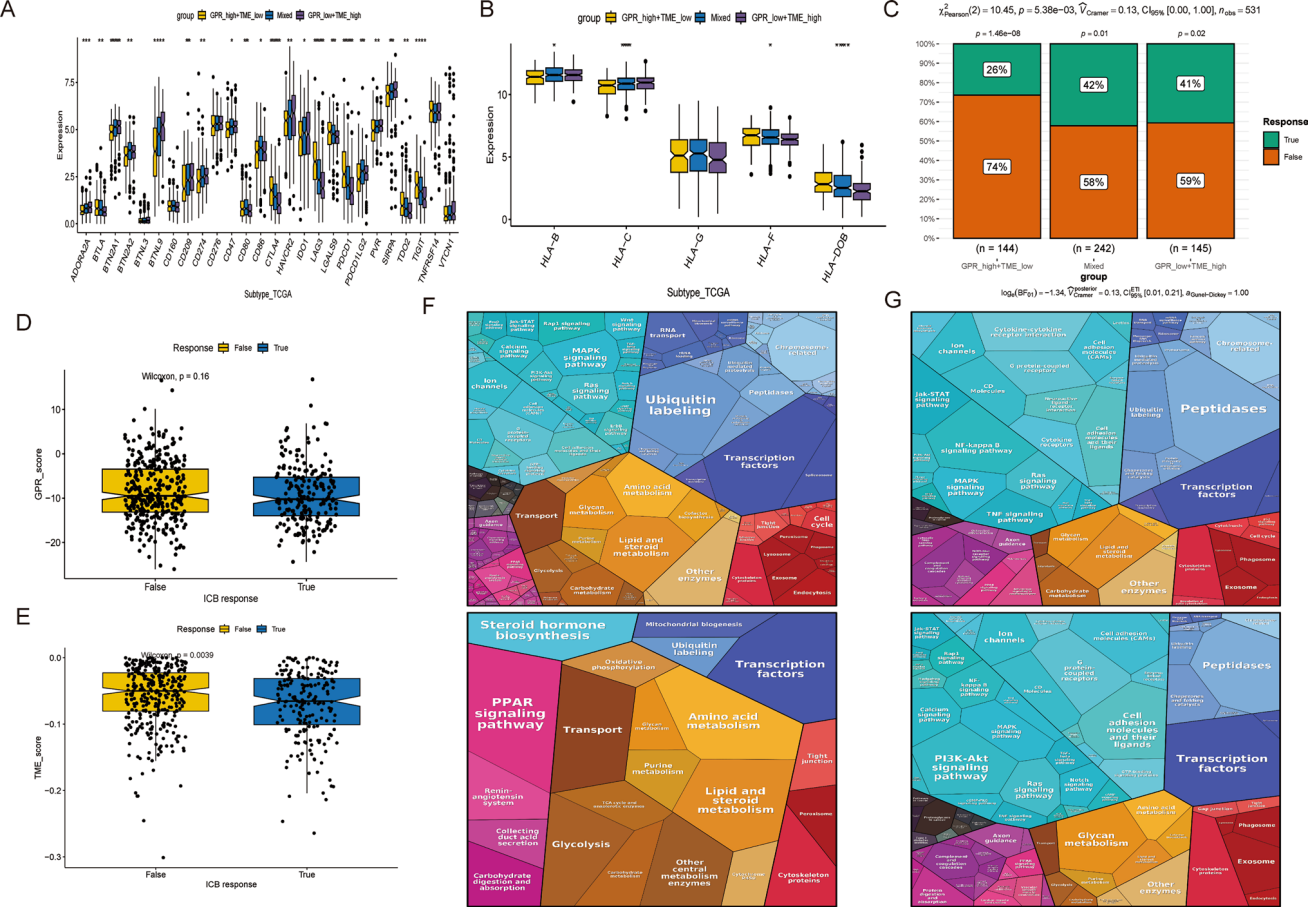
Immunotherapy effect and pathway analysis. **A**, **B** Each immunoregulator was significantly different between disease and control groups **C** Proportion of immune response between different renal clear cell carcinoma subgroups. **D**, **E** GRPs/TME scores of high and low immune response disparity groups. **F**, **G** Fragmentation plots of genes associated with immune disparity and prognostic disparity among different renal clear cell carcinoma subgroups

Grouped according to GRPs and TME gene scores and presented vital differential genes between groups in box plots (Fig. 7A, B)—the differential expression of critical genes in tumor tissues and controls and predictive analysis. In addition, we constructed immune response True and False groups according to immune response gap grouping, in which the proportion of the True group was higher than the Mixed group in the GRPs low group + TME high group. The proportion of True in the Mixed group was higher than GRPs high group + TME low group (Fig. 7C). the GPRs score and TMEa score of the True group were higher than False group (Fig. 7D, E). The fragmentation plot shows the proportion of metabolic pathways among the four subgroups, where the functions and pathways of genes associated with high GRPs + high TMEs mainly included peptidases, NF-kappa B signaling pathway, Cytokine–cytokine receptor interaction, G protein-Functions and pathways of genes associated with GRPs high + TMEs low MAPK signaling pathway, Pl3K-Akt signaling pathway, Transcription., Functions and pathways of genes associated with GRPs low + TMEs high Ubiquitin labeling, MAPK signaling pathway, Calcium signaling pathway. Amino acid metabolism, PPAR signaling pathway, Carbohydrate digestion, absorption digestion, and absorption (Fig. 7F, G).

#### XCR1 genes knockdown inhibits proliferation, migration, and EMT of renal cancer cells

**Fig. 8:**
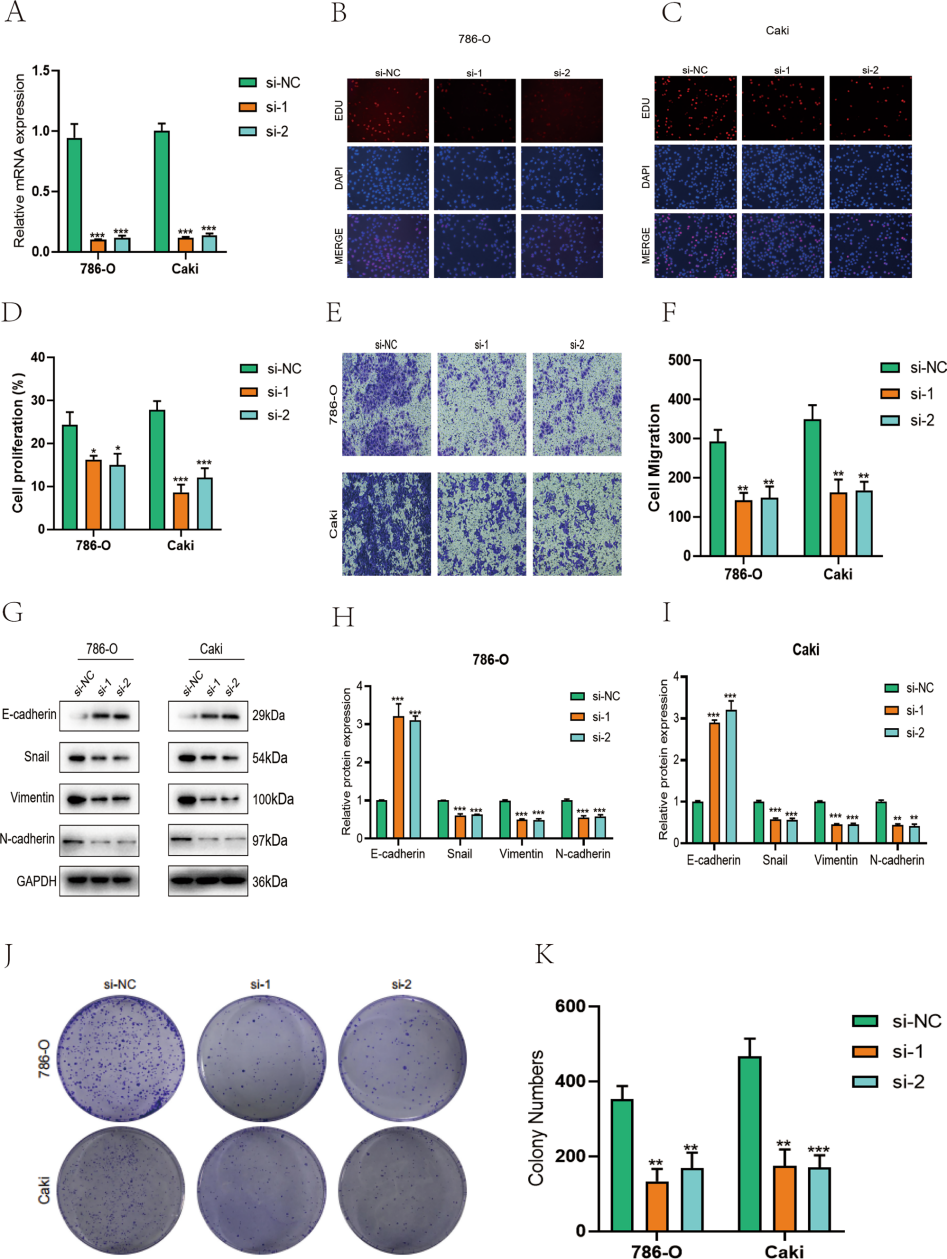
XCR1 genes knockdown inhibits proliferation, migration and EMT of kidney cancer cells. **A** Reduced mRNA expression levels of XCR1 genes after specific siRNA transfection into renal cancer 786-O and CAKI-1 cells. **B**–**D** Knockdown of the XCR1 genes inhibited cell proliferation. **E**, **F** The deletion of the XCR1 genes hindered the migratory ability of renal carcinoma cells, **G**–**I** decreased protein levels of N-calmodulin and Vimentin, increased protein levels of E-calmodulin. **J**, **K** The number of clones was significantly reduced after the knockdown of XCR1 genes in the cells

siRNAs were transfected into kidney cancer 786-O and CAKI-1 cell lines to explore the detailed role of XCR1 genes in oncogenicity. The efficiency of the knockdown of the XCR1 genes was confirmed by RT-qPCR results shown, in which the mRNA expression level of the XCR1 genes was reduced after transfection of specific siRNA into kidney cancer 786-O and CAKI-1 cells (Fig. 8A). These data indicate the high specificity and transfection efficiency of siRNA. In addition, when the XCR1 genes were silenced intracellularly, EDU-positive cells were also reduced, which further confirmed that the knockdown of XCR1 genes inhibited cell proliferation (Fig. 8B, D). In addition, cell migration was detected by Transwell assay. The results showed that the deletion of the XCR1 genes hindered the migration ability of cancer cells, as the number of migrating cells was reduced in the transfected group (Fig. 8E, F). Meanwhile, a lack of XCR1 genes inhibited EMT in kidney cancer, as evidenced by decreased protein levels of N-calmodulin and Vimentin and increased E-calmodulin (Fig. 8G, I). Subsequently, clone formation experiments showed that the number of clones was significantly reduced after the XCR1 genes were knocked down in the cells (Fig. 8J, K). The results above suggest that XCR1 genes downregulation inhibited renal cancer cell proliferation, migration, and EMT.

## Discussion

Biomarkers are essential in evaluating the therapeutic efficacy and prognosis of tumors and can be an essential component of precision medicine [23–25]. Many tumor prognosis-related markers have been discovered with the help of technologies such as high-throughput sequencing and microarrays. These can help evaluate the therapeutic effects of tumors and enhance the survival prognosis of patients [26–28]. Due to the difficult-to-detect nature of kidney cancer and its insensitivity to standard radiotherapy and chemotherapy, its prognosis is often poor, with up to one-third of patients undergoing partial or radical nephrectomy eventually developing metastatic kidney cancer [29, 30]. G protein-coupled receptors play an essential role in the growth of tumor cells, and high expression of G protein-coupled receptors is associated with tumor cell invasion and pathological staging [31, 32]. In this study, we investigated the prognostic value of G protein-coupled receptors in renal cancer and their possible regulatory mechanisms in renal cancer.

We found 163 genes with strong prognostic correlation with patients with renal clear cell carcinoma by constructing and optimizing a prognostic model using univariate Cox regression analysis. Multifactorial Cox regression analysis of the training set data yielded 21 genes with GRPs as independent predictors, namely CASR, ADGRV1, P2RY8, QRFPR, F2RL3, LGR4, SCTR, GPRC5C, GPR84, ADORA2B, OPN1SW, F2RL1, GABBR1, XCR1, ADGRG1 OR2B6, ADGRF5, PTGDR2, EDNRB, PDGFRL, and OR13A1. The samples in the training group were scored, and their GRPs correlation scores were calculated, with the median score as the cut-off; those with scores more remarkable than the median were the high GRPs group, and those below the score were the low GRPs group. The predictive analysis showed that the high GRPs group had a worse prognosis than the low GRPs group. Using the survfit function to assess those immune cell types associated with prognosis, the results showed that naive B cells, monocytes, and activated dendritic cells were associated with prognosis, and the CIBERSORT method to calculate immune infiltration in TCGA samples was bounded by the median score, and those with scores more remarkable than the median were the TMEs high subgroup. Those below the score were the TMEs low subgroup. The results of the analysis showed that the TMEs high subgroup had a better prognosis than the TMEs low subgroup, indicating that GRPs scores were negatively correlated with patient prognosis and TMEs scores were positively correlated with patient prognosis. To test the reliability of GRPs and TMEs genes in the prognosis of patients with renal clear cell carcinoma, we divided the patients into four gene groups according to the GRPs and TMEs gene expression levels in cancer patients, GRPs high + TMEs high, GRPs high + TMEs low, GRPs low + TMEs high, and GRPs low + TMEs low groups, and the predictive analysis showed that the GRPshigh−TMElow group had the worst prognosis and GRPslow−TMEhigh group had the best prognosis, and the AUCs based on GRPs and TMEs gene expression level prediction models at 3, 5 and 7 years were 0.694, 0.761, and 0.724, respectively, all of which were more significant than 0.6, indicating an excellent predictive efficacy.

Second, based on XENA-TCGA-KIRC downloaded data from patients with renal clear cell carcinoma, package GO enrichment analysis showed GPR_low + TME_High: enrichment in metanephric epithelium development, inhibitory postsynaptic potential Mixed: enriched in response to molecule of bacterial origin, histone ubiquitination, and histone ubiquitination. Histone ubiquitination, interferon alpha production, and hormone regulation of sysTMEic arterial blood pressure. gpr_high + tme_low: enriched in mesenchymal cell differentiation, response to BMP, endothelial cell migration, cardiac muscle cell action potential, and negative regulation of smooth muscle cell proliferation. The correlation heat map showed relatively high expression of CD8 T cell recruiting, NK cell.recruiting, Th1 cell.recruiting, Macrophage recruiting in the GPR_high + TME_low group. CD4 T cell recruiting, Th1 cell recruiting, and Macrophage recruiting were relatively high in the GPR_low + TME_high group.CD4 T cell recruiting, CD8 T cell recruiting, and Monocyte recruiting expression were relatively high. The prediction model results showed that the combination of GRPs low grouping and TME high grouping was the best prognosis type, and the intermediate type was defined as mixed. The prognosis of GRPs low grouping + TME high grouping was better. The results of forest plot analysis showed that GRPs and TME scores were correlated with the prognosis of renal clear cell carcinoma and were repeated in the GSE167573 validation group. The results were consistent with the experimental group.

Single-cell RNA sequencing analysis was applied to analyze the cellular differences between cancerous and normal regional tissues in 2 patients with renal clear cell carcinoma. The single-cell sequencing data were subjected to data normalization, dimensionality reduction analysis, and cluster analysis, and then all cells were classified into 21 cell clusters. They included 7 immune cells clusters such as myeloid, endo, Masticells, Nkcells, CD8-T, Tregs, and Stromal. The GPRs scores of various immune cell clusters were also demonstrated. The results of intercellular communication analysis showed that myeloid, endo, Masticells, Nkcells, CD8-T, Tregs, Stromal, and other cell clusters interacted closely. In addition, PROGENy scores showed that Masticells, Nkcells, CD8-T, and Tregs were positively correlated with the activation of “NFkB, TNFa, JAK-STAT, WNT, EGFR, MAPK, VEGF, PI3K, Estrogen Trail”.

In contrast, myeloid, endo, and Stromal cells were positively correlated with “TGFb, Hypoxia, Androgen, and p53 activation”. In addition, we grouped GRPs and TME genes according to their scores to show essential differential genes between groups in a box plot—a differential expression of critical genes in tumor tissues and controls and predictive analysis. In addition, we constructed immune response True and False groups according to immune response gap grouping, in which the proportion of the True group was higher than the Mixed group in the GRPs low group + TME high group, and the proportion of True in the Mixed group was higher than GRPs high group + TME low group. The GPRs score and TMEa score in the True group were higher than the False group. The fragmentation plot shows the proportion of metabolic pathways among the four subgroups, where the functions and pathways of genes associated with high GRPs + high TMEs mainly included peptidases, NF-kappa B signaling pathway, Cytokine–cytokine receptor interaction, G protein- Functions and pathways of genes associated with GRPs high + TMEs common MAPK signaling pathway, Pl3K-Akt signaling pathway, Transcription, Functions and pathways of genes associated with GRPs low + TMEs high Ubiquitin Amino acid metabolism, PPAR signaling pathway, Carbohydrate digestion, and absorption. The cell culture results showed that when XCR1 genes were silenced intracellularly, EDU-positive cells were also reduced, which further confirmed that the knockdown of XCR1 genes inhibited cell proliferation. In addition, the results of the cell migration assay by Transwell showed that the deletion of the XCR1 genes hindered the migration ability of cancer cells, as the number of migrating cells was reduced in the transfected group. Also, the lack of the XCR1 genes inhibited EMT in kidney cancer, as evidenced by decreased protein levels of N-calmodulin and Vimentin and increased E-calmodulin. Subsequently, clone formation experiments showed that the number of clones was significantly reduced after XCR1 genes were knocked out in the cells. The results above suggest that XCR1 genes downregulation inhibited renal cancer cell proliferation, migration, and EMT.

## Conclusion

This study examined the role of G protein-coupled receptor-associated genes in the prognosis of patients with renal clear cell carcinoma. We used data from TCGA to develop a prognostic prediction model based on G protein-coupled receptor-related genes in survivors of renal clear cell carcinoma patients, which offers hope for the management of renal clear cell carcinoma.

## Data Availability

We sincerely appreciate all members who participated in data collection and analysis. Publicly available datasets were analyzed in this study. This data can be found here: https://www.ncbi.nlm.nih.gov/geo/, with the accession number GSE167573 and GSE152938.
